# Social ecological factors associated with physical activity and screen time amongst mothers from disadvantaged neighbourhoods over three years

**DOI:** 10.1186/s12966-020-01015-5

**Published:** 2020-08-28

**Authors:** Kylie A. Morris, Lauren Arundell, Verity Cleland, Megan Teychenne

**Affiliations:** 1 Geelong, Australia, School of Exercise and Nutrition Sciences, Deakin University, Melbourne Burwood Campus, 221 Burwood Hwy, Burwood, VIC 3125 Australia; 2 Geelong, Australia, Institute for Physical Activity and Nutrition (IPAN), School of Exercise and Nutrition Sciences, Deakin University, Burwood, Australia; 3 Menzies Institute for Medical Research, University of Tasmania, Hobart, Australia

**Keywords:** Physical activity, Sedentary behaviour, Screen time, Mothers, Socioeconomic disadvantage

## Abstract

**Background:**

Mothers from socioeconomically disadvantaged neighbourhoods are at elevated risk of physical inactivity and high levels of screen time. Yet, little is known regarding the social ecological factors that are longitudinally associated with physical activity and screen time in this target group, and whether the age of their children impacts these relationships. This study aimed to longitudinally examine the social ecological factors associated with physical activity and screen time amongst mothers living in socioeconomically disadvantaged neighbourhoods, and whether these differed according to their child’s age.

**Methods:**

Data were from 895 mothers living in socioeconomically disadvantaged neighbourhoods (mean age 36.7 years) at baseline and three-year follow-up. Mothers self-reported weekly discretionary physical activity (leisure-time, LTPA; transport-related, TRPA) and screen time durations. Linear regression models assessed associations between five intrapersonal, three social and five physical environmental factors and LTPA, TRPA and screen time, adjusting for confounding factors, clustering by neighbourhood and baseline variables. Interaction analysis was conducted for age of children (younger and older children (*n* = 442) and mothers with older children (aged 5–12 years) only (*n* = 453).

**Results:**

In adjusted models, all intrapersonal factors (self-efficacy, enjoyment, outcome expectations, behavioural intentions and behavioural skill), social support from friends, neighbourhood cohesion and number of televisions were longitudinally associated with LTPA amongst all mothers. Interaction models showed that findings were generally consistent across groups (i.e., those with both younger and older children compared to those with older children only), with three exceptions. Physical activity enjoyment and social support from family were associated with LTPA only among mothers with older children. Neighbourhood cohesion was associated with screen time only amongst mothers with both younger and older children. No associations were detected for TRPA.

**Conclusion:**

Intrapersonal, social and physical environmental factors were longitudinally associated with mother’s LTPA, whilst neighbourhood cohesion was longitudinally associated with screen time behaviours amongst mothers. Interventions aimed at increasing LTPA amongst mothers (particularly those from socioeconomically disadvantaged neighbourhoods) may need to target all domains of the social ecological model and may require some tailoring according to the age of children. Further work is needed to identify longitudinal associations with screen time and TRPA in this population group.

## Background

Physical inactivity is the fourth leading risk factor for mortality globally, contributing annually to over three million preventable deaths [1]. Physical activity has been linked to reduced risk of physical [2] and mental [3] health conditions. Despite this, more than half the population in countries such as Australia, the US and the UK [4–6] do not meet current physical activity guidelines, with women less physically active than men [5, 7]. As women enter into motherhood, there is a decline in physical activity [8–11], where 85% of women have reported they were more active before having children [10]. Furthermore, first-time mothers were 1.8 times more likely to be inactive compared to women without children 4 years after motherhood [11]. This indicates that the negative changes in mother’s health behaviours are still occurring many years after the birth of their child.

Engaging in high levels of sedentary behaviour (e.g., TV viewing, computer use, occupational sitting) has been linked to increased risk of physical [2] and mental health conditions [3, 12], independent of physical activity [13]. Yet women spend on average 39 h per week engaged in sedentary behaviour [4], with about half (20 h) of this time in television viewing [4]. Higher rates of sedentary behaviour have been seen in women (52%), compared to men (42%) [4], with increases in sedentary behaviour escalating during motherhood [11].

Internationally [6] certain sub-groups are at greater risk of physical inactivity and sedentary behaviours, including women (particularly mothers) and those living in socioeconomically disadvantaged neighbourhoods. Specifically, mothers from socioeconomically disadvantaged neighbourhoods participate 38 min per week less physical activity than mothers living in less disadvantaged neighbourhoods [11]. In order to inform targeted and effective intervention strategies it is important to understand factors that may influence these behaviours. The social ecological model [14] has been used in behavioural research to examine the factors that may influence physical activity and/or sedentary behaviours. This model recognises multiple levels of influence on behaviour, encompassing intrapersonal (motivation, self-efficacy, ability and goal setting), social (support from family/spouse and friends/work colleagues) and physical environmental (recreational facilities, personal safety and likeable neighbourhoods) constructs [14, 15].

Overall, little research has examined the longitudinal factors associated with mothers’ physical activity and sedentary behaviour across the multiple levels of the social ecological model, nor have studies acknowledged that these longitudinal associations may differ between mothers with younger children and mothers with older children [16, 17]. Previous research has shown factors associated with mothers’ physical activity levels are intrapersonal (e.g., time constraints [18–21], being tired or fatigued [18, 22]), from the social environment (e.g., support from family, friends and work colleagues [20, 22], childcare access [18, 21, 22]) and from the physical environment (e.g., access to facilities [20, 23], cost of facilities [20, 24]). Regarding mothers’ sedentary behaviours far fewer studies have examined these factors. However, amongst women generally, intrapersonal (e.g., leisure-time physical activity [25, 26], weight status [25, 27]) and physical environmental factors (e.g., area of residence [25, 28]) have been associated with sedentary behaviour. Few studies have examined associations between social factors and women’s sedentary behaviour levels, highlighting a major gap in the research. Most existing studies are cross-sectional [18, 23] meaning they are unable to determine the direction of relationships, and very few included mothers from socioeconomically disadvantaged neighbourhoods [26, 29].

Therefore, the aim of this study was to longitudinally examine the intrapersonal, social and physical environmental factors associated with engaging in physical activity and screen time amongst mothers (with children aged 0–12 years) living in socioeconomically disadvantaged neighbourhoods. Additionally, this study examines whether any associations differ according to the age of the children.

## Methods

Findings are reported according to STROBE guidelines [30]. Self-report data in 2007–08 and 2010–11 was collected, as part of a longitudinal study: the Resilience for Eating and Activity Despite Inequality [READI] study [9, 31]. The READI study was approved by the Deakin University Human Research Ethics Committee and all participants provided written consent. A detailed description of the study is provided elsewhere [31].

### Sample and data collection

**Fig. 1:**
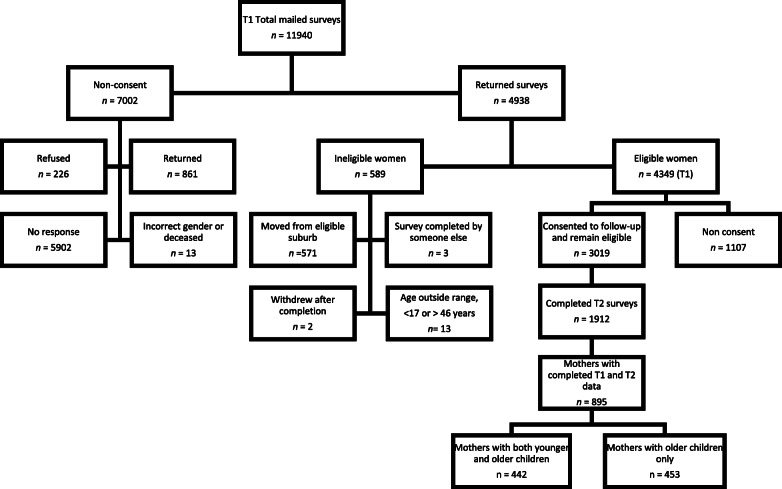
Flow-chart of participants in the READI study at baseline (2007–08) and T2 (2010–11) (mother participants)

An area-level indicator of socioeconomic disadvantage (the Index of Relative Socio-economic Disadvantage [SEIFA]) determined by the population census that considers factors such as income, employment and education [32], was used to classify all Victorian neighbourhoods. Neighbourhoods in the bottom third were considered ‘disadvantaged’. From this sample, women (aged 18–45 years) residing in 40 urban and 40 rural socioeconomically disadvantaged neighbourhoods were randomly selected to participate. The Australian Electoral Roll was used to randomly choose 150 women from each of the 80 neighbourhoods, with a total sampling pool of 11,940 women aged 18–45 years invited to participate. A total of 4938 completed surveys were completed (proportion of responses = 45%). After applying eligibility criteria there were 4349 respondents at baseline (T1) (refer Fig. 1). A follow-up survey was sent 3 years after baseline (T2: 2010–11) to women who indicated they were happy to be re-contacted about future research (*n* = 3019). Completed T2 surveys were collected from 1912 respondents (63% of eligible participants). Women were excluded from the current study if they did not have a child aged ≤12 years living at home at baseline and did not have complete independent and outcome variable data at T1 and T2 (*n* = 895 included; *n* = 1017 excluded).

### Outcome variables

#### Physical activity

Participants reported their frequency and duration of physical activity over the past week across four domains (occupation, transport, leisure, domestic) using the International Physical Activity Questionnaire – long form (IPAQ-L) [33]. A reliable (pooled *r* = 0.81) and valid (mean *p* = 0.30) tool for examining adult population levels of physical activity. For this study, only leisure-time and transport-related physical activity were investigated since these activities are typically discretional and are more likely to be targeted in physical activity interventions for mothers. For both the leisure-time and transport domains, time (frequency x duration) was summed to calculate the total weekly time spent (in minutes) being active within these domains. The data was truncated according to an established protocol [34].

### Screen time

Participants reported time spent in screen time (i.e., combined computer and television use) over the past week. Valid and reliable measures (Intraclass Correlation Coefficients [ICC]) were used to record separately time spent sitting watching television (ICC = 0.82) and sitting using the computer (ICC = 0.62) [35]. An ICC 0.40–0.75 represents a fair to good agreement and > 0.75 represents excellent agreement [35]. Both television viewing and computer use duration were computed (sum of [weekday × 5] + [weekend day × 2]). The data was truncated using an adapted protocol [34]. Weekly screen time’ duration was calculated by adding the weekly duration for the variables ‘television viewing’ and ‘computer use’.

### Independent variables

Thirteen independent variables encompassing three constructs of the social ecological model (i.e., intrapersonal, social, physical environmental) were included in the analysis. These items were from previously developed studies and have been tested for reliability [36–42]. Details of these constructs and questions included are provided in Table 1. For variables with more than one item (e.g., neighbourhood walkability), items were summed to calculate a total score for that variable.

**Table 1:** Survey items used to examine intrapersonal, social and physical environmental factors in the READI study

Variable	Likert scale/response options	Internal reliability (α)^a^	Data management	Questions used to assess variable
*Intrapersonal factors*				
Behavioural skills [36]	4-point: 1 (never), 2 (once or twice), 3 (weekly), 4 (more than once/week)	0.83	Sum 2 items	How many times in the past month did you: ‘Set a goal for how much physical activity you would like to do?’, ‘Plan particular days on which you would do physical activity?’
Behavioural intentions [36]	7-point: 1 (very unlikely), 7 (very likely)	N/A	dichotomise	Assuming that you tried to do physical activity over the next 2 weeks, how likely or unlikely is it that you would actually stick to this?
Outcome expectancies [38]	4-point: 1 (no reason at all), 4 (very important reason)	0.79	Sum 6 items	How important do you think these reasons are for being physically active? ‘Health’, ‘Appearance’, ‘Weight’, ‘Feeling fit’, ‘Relaxation’, ‘Stress relief’
Enjoyment [37]	7-point: 1 (least enjoyable), 7 (most enjoyable)	0.95	Sum 6 items	Feelings about physical activity: ‘I love it/I hate it’, ‘I feel interested/I feel bored’, ‘I find it pleasurable/I find it unpleasurable’, ‘I find it energising/I find it tiring’, ‘It makes me happy/It makes me depressed’, ‘I feel good physically while doing it/I feel bad physically while doing it’
Self-efficacy [39]	5-point: 1 (strongly agree), 5 (strongly disagree)	0.82	Sum 5 items	I am confident that I could do physical activity even when: ‘I am tired’, ‘I am in a bad mood’, ‘I feel I don’t have time’, ‘I am on holiday’, ‘it is raining’
*Social factors*				
Social support from family/spouse [41]	5 -point: 1 (never), 5 (very often)	0.76	Sum 2 items	During the past year, how often did members of your family: ‘Do physical activity with you?’, ‘Encourage you to be physically active?’
Social support from friends/work colleagues [41]	5-point: 1 (never), 5 (very often)	0.69	Sum 2 items	During the past year, how often did friends or work colleagues: ‘Do physical activity with you?’, ‘Encourage you to be physically active?’
Childcare	1 (yes), 2 (no), 3 (N/A/I don’t have children)	N/A	dichotomise	If you wanted to do any physical activity without your children, do you have access to childcare either at a childcare centre, a partner/family member or a friend?
*Physical environment factors*				
Neighbourhood walkability [40]	5-point:1 (strongly agree), 5 (strongly disagree)	0.80	Sum 7 items	‘My neighbourhood offers many opportunities to be physically active’, ‘Local sports clubs and other facilities in my neighbourhood offer many opportunities to get exercise’, ‘It is pleasant to walk in my neighbourhood’, ‘The trees in my neighbourhood provide enough shade’, ‘In my neighbourhood it is easy to walk places’, ‘I often see other people walking in my neighbourhood’, ‘I often see other people exercising (e.g., jogging, bicycling, playing sports) in my neighbourhood’
Neighbourhood aesthetics [40]	5-point: 1 (strongly agree), 5 (strongly disagree)	0.76	Sum 5 items	‘There is a lot of rubbish on the street in my neighbourhood’, ‘There is a lot of noise in my neighbourhood”, In my neighbourhood the buildings and homes are well-maintained’, ‘The buildings and homes in my neighbourhood are interesting’, ‘My neighbourhood is attractive’
Personal safety [40]	5-point: 1 (strongly agree), 5 (strongly disagree)	0.85	Sum 3 items	‘I feel safe walking in my neighbourhood, day or night’, ‘Violence is not a problem in my neighbourhood’, ‘My neighbourhood is safe from crime’
Neighbourhood cohesion [42]	5-point: 1 (strongly agree), 5 (strongly disagree)	0.83	Sum 7 items	‘People in this neighbourhood can be trusted’, ‘This is a close-knit neighbourhood’, ‘People around here are willing to help their neighbours’, ‘People in this neighbourhood generally don’t get along with each other’, ‘People in this neighbourhood do not share the same values’
Number of televisions per household	5-point: 0 (None), 4 (four or more)	N/A	dichotomise	‘How may televisions do you have in your house?’

Notes: ^a^ Cronbach’s alpha, *N/A* Non-applicable

### Sociodemographic characteristics

Participants reported their age, highest educational level (low = <Year 12; medium = Year 12, certificate/trade/diploma; high = tertiary), number and age of children living at home (< 2 years, 2–4 years, 5–12 years). Weight status (calculated as body mass index [BMI]) from reported height and weight data (kg/m2) using established methods [43] and categorised as: not overweight (BMI < 25 kg/m^2^), overweight (BMI 25–29.9 kg/m^2^) or obese (BMI ≥30 kg/m^2^) [44]).

### Missing data

Participants were excluded where they did not contain complete independent or outcome variables for the specific analyses of interest. Variables with missing baseline data included; age (0.22%), education (0.56%), household income (7.04%), employment status (1.79%), paid work status (1.90%), marital status (0.11%), BMI status (4.47%), physical activity enjoyment (1.56%), outcome expectations (0.78%), self-efficacy (0.34%), childcare (0.11%), physical activity family support (0.34%), physical activity friend support (0.22%), screen time family support (0.45%), screen time friend support (0.45%), neighbourhood cohesion (0.34%), aesthetics (0.67%), safety (0.22%), walkability (0.78%), leisure time physical activity (3.02%), transport related physical activity (3.02%), screen time activity (7.93%).

### Statistical analysis

Data was analysed in Stata version 15. Descriptive statistics (mean and standard deviation for continuous data and frequencies and percent for categorical data) were used to explore the sociodemographic characteristics of the sample of mothers at baseline (Table 2). Medians and inter-quartile ranges were used to describe the behavioural characteristics of mothers at baseline and follow-up (Table 3) due to the non-normal distribution of the physical activity and sedentary behaviour data. To address the studies aims, crude (adjusted only for baseline outcome value and clustering by neighbourhood) and adjusted (adjusted for baseline outcome value, clustering by neighbourhood and potential confounders of age, education, number of children, weight status and number of paid hours worked) linear regression models were used. These models were used to examine the longitudinal association between the independent variables (intrapersonal, social and environmental factors) reported at T1 and the outcome variables (i.e., leisure-time physical activity, transport related physical activity or screen time) reported at T2. Confounders were selected based on being theoretically associated with both outcome and independent variables. Separate linear regression models were run for each of the outcome variables (i.e., leisure-time activity, transport-related activity, and screen time) against each of the thirteen independent variables.

**Table 2:** Sociodemographic characteristics of mothers at baseline

Sociodemographic Characteristics	*n*	Mean (±SD)/ %
***Age*** *(mean years)*	893	36.7 (±6.2)
***Education***		
Low - < Year 12	207	23
Medium – Year 12/trade/diploma	429	48
High – completed tertiary education	254	29
***Household Income***		
Low - $0–699/wk	612	74
Medium - $700–1499/wk	146	18
High - $1500+/wk	14	2
Not disclosed	60	7
***Employment Status***		
Working full-time	162	18
Working part-time	331	38
Not currently working (paid work)	386	44
***Paid Work Status***		
Not currently working (paid work)	291	33
< 25 h/wk	307	35
25–40 h/wk	222	25
> 40h hours/wk	58	7
***Marital Status***		
Married/defacto relationship	771	86
Separated/divorced/widowed	77	9
Never married	46	5
***BMI Status***		
Not overweight	454	53
Overweight	227	72
Obese	174	20
***Country of Birth***		
Australia	829	93
Other	66	7
***Age of children living at home***		
Children aged 0–4 years	442	49
Children aged 5–12 years	453	51

Notes; *BMI* Body mass index

**Table 3:** Physical activity and sedentary behaviour characteristics of mothers at baseline and follow-up (minutes/week)

Behavioural characteristics	T1			T2		
	n	Median (Q25, Q75)	IQR	n	Median (Q25, Q75)	IQR
Total LTPA	868	100 (0, 240)	240	892	120 (120, 270)	270
Total TRPA	868	90 (0, 180)	180	883	60 (0, 180)	180
Screen time	824	1470 (960, 2280)	1320	895	1680 (1080, 2520)	1440

Notes: *LTPA* Leisure-time physical activity, *TRPA* Transport related physical activity, *Q25* 25th quartile, *Q75* 75th quartile, *IQR* Inter quartiles range

Secondly, to detect an interaction between mothers according to the age of the child at baseline, mothers were categorised into two groups; mothers with both younger and older children (includes those with a least one child aged 0–5 years) and mothers with older children only (includes those only with children aged 5–12 years). Linear regression models were used to examine associations between the independent and outcome variables of the two mothers’ groups. This involved adding an interaction term (age group of child*outcome) into the models. Where there was a significant interaction (*p* < 0.05), further regression models were stratified to examine relationships between the outcome and independent variables separately for each mothers group (those with both younger and older children and mothers with only older children only). The residual and predicted values met the assumptions of homoscedasticity. The distributions of each outcome variable were tested for normality and subsequently transformed to be as close as possible to a normal distribution using a square root transformation. Collinearity was assessed by examining the variance inflation factor [VIF] values and these were acceptably low (< 2).

## Results

### Sociodemographic characteristics of the sample

Sociodemographic characteristics of the sample consisted of 893 mothers (mothers *n* = 442 with both younger and older children and mothers *n* = 453 with older children only) and are presented in Table 2.

### Physical activity and screen time

Mothers reported a median of 1.7 h of leisure-time physical activity per week at baseline and 2 h at follow-up (Table 3). Similarly, mothers reported a median of 1.5 h per week of transport related physical activity at baseline and 1 h at follow-up. Mothers reported a median of 24.5 h per week of screen time activities at baseline and 28 h at follow-up. Comparisons between baseline behavioural outcomes between participants with and without complete T1 and T2 data showed no significant differences.

### Longitudinal associations with leisure-time physical activity

Linear regression analysis of the intrapersonal, social and physical environmental factors associated with mother’s leisure-time physical activity are shown in Table 4. After adjusting for confounders (model 2), amongst all mothers, all five intrapersonal factors (physical activity enjoyment, outcome expectations, self-efficacy, behavioural intentions and behavioural skills) at baseline were positively associated with leisure-time physical activity at follow-up. A significant interaction (*p* < 0.05) between the child age group and leisure-time physical activity was found for physical activity enjoyment. Further investigation showed that baseline physical activity enjoyment was positively associated with leisure-time physical activity at follow-up among mothers with older children (β 0.27, 95% CI 0.15,0.39) but not mothers with both younger and older children (β 0.07, 95% CI -0.08,0.22).

**Table 4:** Linear regression analysis of the social ecological factors associated with mother’s leisure time physical activity

	Model 1^a^		Model 2^b^	
	β	(95%CI)	β	(95%CI)
*Intrapersonal*				
PA enjoyment	**0.20**	0.11,0.28***	**0.19**	0.09,0.28***
Outcome expectations	**0.33**	0.16,0.50***	**0.28**	0.10,0.46*
Self-efficacy	**0.41**	0.27,0.55***	**0.43**	0.29,0.57***
Behaviour Intentions	**0.76**	0.37,1.16***	**0.69**	0.27,1.11*
Behaviour skill	**0.74**	0.43,1.04***	**0.72**	0.39,1.06***
*Social*				
Childcare	**1.00**	0.02,1.98*	0.44	−0.49,1.36
Social support: family/spouse	0.23	−0.03,0.49	0.27	− 0.01,0.53
Social support: friends/work colleagues	**0.54**	0.28,0.80***	**0.56**	0.29,0.84***
*Environment*				
Cohesion	**0.35**	0.13,0.57*	**0.34**	0.09,0.58*
Safety	0.06	−0.15,0.28	0.04	− 0.17,0.24
Aesthetics	**0.25**	0.01,0.49*	0.22	−0.02,0.46
Walkability	**0.18**	−0.53,0.31*	0.14	−0.00,0.28
No. of TVs^c^	−0.29	−0.80,0.22	**− 0.69**	−1.23,-0.16*

Notes: * *p* = <.05, ** *p* = ≤.001, *** *p* = .0001; β coefficient, *95% CI* Confident intervals

^a^adjusted for baseline variable and clustering by neighbourhoods

^b^adjusted for age, education, number of children, BMI, baseline variable and clustering by neighbourhoods; *PA* Physical activity, ^c^Number of television sets per household

Within the social domain, having social support from friends/work colleagues at baseline was positively associated with leisure-time physical activity at follow-up for all mothers in the adjusted model (Table 4). A significant interaction between the child age group and leisure-time physical activity was found for having support from family/spouse members. Baseline support from family/spouse members was positively associated with leisure-time physical activity at follow-up amongst mothers with older children only (β 0.68, 95% CI 0.27,1.10) but not mothers with both younger and older children (β − 0.20, 95% CI -0.60,2.43). No other social factors or child age group interactions were associated with mother’s leisure-time physical activity at follow-up.

Neighbourhood cohesion was positively associated with mother’s leisure-time physical activity at follow-up, whilst number of televisions was negatively associated with leisure-time physical activity at follow-up among all mothers. No interactions with the age group of the children were detected.

### Longitudinal associations with transport-related physical activity

There were no significant associations between any intrapersonal, social or physical environmental factors at baseline and mother’s transport-related physical activity at follow-up (see Table 5). No significant interactions with the child age group were detected.

**Table 5:** Linear regression analysis of the social ecological factors associated with mother’s transport related physical activity

	Model 1^a^		Model 2^b^	
	β	(95%CI)	β	(95%CI)
*Intrapersonal*				
PA enjoyment	0.04	−0.03,0.11	0.03	−0.06,0.10
Outcome expectations	0.08	−0.08,0.25	0.10	−0.08,0.28
Self-efficacy	0.08	−0.07,0.22	0.06	−0.09,0.21
Behaviour Intentions	0.13	−0.23,0.48	0.18	−0.21,0.56
Behaviour skill	0.15	−0.12,0.41	0.24	−0.04,0.52
*Social*				
Childcare	−0.17	−1.10,0.76	−0.17	−1.13,0.79
Social support: family/spouse	0.12	−0.12,0.35	0.12	−0.13,0.36
Social support: friends/work colleagues	0.10	−0.12,0.33	0.10	−0.15,0.34
*Environment*				
Cohesion	−0.03	−0.22,0.17	− 0.05	−0.27,0.17
Safety	−0.02	−0.19,0.16	− 0.04	−0.22,0.13
Aesthetics	−0.09	−0.24,0.67	− 0.02	−0.19,0.15
Walkability	0.06	−0.08,0.19	0.07	−0.08,0.21
No. of TVs^c^	−0.54	−1.18,0.09	−0.18	− 0.81,0.44

Notes: β = coefficient; ^a^adjusted for baseline variable and clustering by neighbourhoods; ^b^adjusted for age, education, number of children, weight status, baseline variable and clustering by neighbourhoods; *PA* Physical activity; ^c^Number of television sets per household

### Longitudinal associations with screen time

There were no significant associations between any baseline intrapersonal, social or physical environmental factors and mother’s screen time at follow-up (Table 6). However, a significant interaction (*p* < 0.05) between child age group and screen time and screen time for neighbourhood cohesion was detected. High levels of neighbourhood cohesion at baseline were longitudinally associated with less screen time at follow-up amongst mothers with both younger and older children (β − 0.67, 95% CI -1.06,-0.28) but not among mothers with older children only (β 0.10, 95% CI -0.42,0.62).

**Table 6:** Linear regression analysis of the social ecological factors associated with mother’s screen time

	Model 1^a^		Model 2^b^	
	β	(95%CI)	β	(95%CI)
*Intrapersonal*				
PA enjoyment	**−0.11**	− 0.20,− 0.01*	-0.01	−0.13,0.11
Outcome expectations	**−0.24**	−0.46,-0.02*	− 0.14	−0.37,0.08
Self-efficacy	**−0.25**	−0.44,-0.06*	− 0.17	−0.37,0.26
Behaviour Intentions	**−0.49**	−0.98,-0.01*	− 0.27	−0.79,0.26
Behaviour skill	−0.03	−0.44,0.37	0.06	−0.37,0.48
*Social*				
Childcare	1.08	−0.25,2.41	0.73	−0.71,2.17
Social support: family/spouse	**−0.37**	−0.70,-0.04*	− 0.26	−0.61,0.09
Social support: friends/work colleagues	0.01	−0.36,0.37	0.08	−0.32,0.48
*Environment*				
Cohesion	**−0.33**	−0.63,-0.03*	− 0.29	−0.64,0.05
Safety	−0.15	−0.46,0.16	− 0.15	−0.50,0.20
Aesthetics	−0.15	−0.45,0.15	− 0.13	−0.46,0.21
Walkability	−0.09	−0.26,0.08	− 0.07	−0.24,0.11
No. of TVs^c^	**1.12**	−0.23,2.02*	0.60	−0.42,1.62

Notes: * *p* = <.05; ** *p* = ≤.001; *** *p* = <.0001; β = coefficient; ^a^adjusted for baseline variable and clustering by neighbourhoods; ^b^adjusted for age, education, number of children, weight status, baseline variable and clustering by neighbourhoods; *PA* Physical activity, *SB* Sedentary behaviour; ^c^Number of television sets per household

## Discussion

This study identified several social ecological factors that were longitudinally associated with physical activity and screen time amongst mothers living in socioeconomically disadvantaged neighbourhoods, although longitudinal associations differed according to the domain of physical activity, as well as the age of the mother’s children.

Amongst all mothers, all five intrapersonal factors were longitudinally associated with higher levels of leisure-time physical activity. Mothers were more likely to participate in leisure-time physical activity if they enjoyed doing it (particularly amongst mothers with older children only), had outcome expectations, greater self-efficacy, behavioural intentions or behavioural skill. These relationships are consistent with previous cross-sectional research amongst mothers [18, 21, 22], socioeconomically disadvantaged groups [18, 20, 45] and adults in general [46, 47]. These findings may assist in the development of intervention strategies targeting leisure-time physical activity of mother’s from socioeconomically disadvantaged neighbourhoods. Further, findings suggested that having a friend or colleague to exercise with and family support was associated with higher levels of leisure time physical activity, consistent with previous research [21, 48–50]. Therefore, future programs may include mother’s yoga classes and women only sport [18] to promote enjoyment and companionship.

This study found that higher levels of neighbourhood cohesion were longitudinally associated with higher levels of mother’s leisure-time physical activity, which has previously been shown to be an important factor in physical activity participation amongst women [51, 52] and in particular mothers (children aged 4–10 years living at home) [53]. Further, more televisions in the home were longitudinally associated with lower levels of leisure-time physical activity for mothers at follow-up. To our knowledge, no previous research has investigated the relationship between the number of televisions per household and physical activity amongst mothers or women in general. However, in youths [54, 55] (aged 6–18 years) having more televisions in the house was associated with more time watching television, which then led to lower levels of physical activity. Although further research on the physical environmental factors associated with physical activity amongst mothers is needed, these findings may suggest that future interventions to promote mother’s physical activity should consider targeting reducing the number of televisions in the house and enhancing social cohesion in the neighbourhood, such as implementing community-based activities and promoting social engagement. The lack of association for all other environmental variables suggests that individual and social factors may be particularly influential on mother’s leisure-time physical activity or other environmental measures (e.g., proximity to local shops/cafes) that were not examined in this project may be associated with leisure-time physical activity.

No significant associations were found between any potential independent variables and transport-related physical activity. Evidence suggests that transport-related physical activity is strongly influenced by the physical and neighbourhood environment [23, 56, 57]. Therefore, mother’s participation in this type of physical activity may be more influenced by factors that were not captured in this study, such as proximity to local shops [23], important destinations (e.g., cafes, parks) and services (e.g., doctors, library), access to dedicated cycles and walking lanes and volume of traffic on roads [56, 57]. Transport-related physical activity may also be influenced by the volume of mothers’ child-oriented tasks [58–60] such as having to pick up children after school and take them to after-school activities, shopping or other routine tasks. Previous studies have found mothers find it more convenient to drive to do these errands than partake in active transport [56, 61]. Future initiatives may include ensuring ‘park and ride/walk’ facilities near schools/childcare centres allowing mothers to drive to school, then walk/cycle to work or other errands. An added benefit of this may be in creating safe school environments, which might encourage more children to walk/cycle to school with their mothers.

The current study found higher levels of neighbourhood cohesion were associated with lower levels of screen time amongst mothers with both younger and older children. Mothers within this age group may be accessing local community activities (e.g., playgroup, library story-time and mothers’ group) making them feel more secure and connected within their neighbourhood, which then displaces time spent in screen time activities. No previous studies have investigated the link between neighbourhood cohesion and screen time and thus further research is warranted to explore and confirm these findings. No other environmental factors were associated with screen time, and nor were any intrapersonal or social factors. A potential reason may be that the measure of total screen time did not capture time spent in different sedentary behaviours (e.g., television viewing vs computer use vs tablet use) or distinguish between discretionary (e.g., television viewing) and non-discretionary (work-related computer usage) screen time which may have different longitudinal associations. Further, many independent variables measured were physical activity-related (e.g., self-efficacy, behavioural intentions, enjoyment of physical activity, behavioural skill or outcome expectations), rather than targeting screen time specifically.

Limitations of this study should be considered. Firstly, the use of self-report measures may be subject to recall difficulties and response biases. Secondly, selection bias could be present due to a modest response rate (45%) and drop-out/loss of follow-up between T1 and T2 sample; however, comparisons between mothers with baseline data only and mothers with baseline and T2 data showed no significant difference in behavioural outcomes. Thirdly, although all respondents lived in socioeconomically disadvantaged neighbourhoods, baseline data suggest a proportion of this sample would not be considered socioeconomically disadvantaged based on individual-level socioeconomic characteristics (e.g., 29% had tertiary education). Therefore, under-sampling of extremely disadvantaged mothers is likely to have occurred and generalisation to all mothers from disadvantaged neighbourhoods may not be possible. Fourth, additional factors not measured in the current analysis may impact physical activity and sedentary behaviours amongst this population group (e.g., weather, distance to local shops, work etc.) and require further investigation. Finally, the multiple models tested could potentially increase the probability of spurious findings. However, we present findings for all pre-specified analyses, provide lower and upper confidence intervals for all estimates, and present exact *p*-values.

A key strength of this study is the large sample size of an understudied and difficult to reach population, which allowed for adjustment of important confounding factors. Further, the longitudinal study design allowed the direction of relationships between to be determined, which builds on the primarily cross-sectional research to date. This study was able to assess intrapersonal, social and physical environmental factors that encompass the social ecological model [14], many of which have not been previously examined in the same sample.

## Conclusion

This study identified several factors longitudinally associated with greater leisure-time physical activity (i.e., enjoyment of physical activity and having support from family/spouse) and less screen time (neighbourhood cohesion) among mothers living in disadvantaged neighbourhoods. Longitudinal associations differed according to the domain of physical activity, as well as the age of the mother’s children. Independent variables were primarily associated with leisure-time physical activity and within the intrapersonal domain of the social ecological model suggesting behaviour specific intervention strategies are required. Overall, the findings of this study may help develop targeted interventions and programs aimed at improving mother’s physical activity participation and lowering their screen time levels, particularly amongst those living in socioeconomically disadvantaged neighbourhoods.

## Data Availability

The datasets analysed during the current study are not publicly available due to ethical restrictions (participants have not consented to the use of their data for purposes other than those for which they originally consented). Should a researcher request the data for a particular purpose, an ethically compliant dataset may be made available via the senior author upon approval by the Deakin University Human Research Ethics Committee. Requests can be emailed to: research-ethics@deakin.edu.au
